# Understanding the differential effects on employment of a community wealth building programme in England: a difference-in-differences study

**DOI:** 10.1136/jech-2024-223499

**Published:** 2025-05-16

**Authors:** Tanith C Rose, Konstantinos Daras, Mick McKeown, Tom Lloyd Goodwin, Julian Manley, Benjamin Barr

**Affiliations:** 1 Department of Public Health, Policy and Systems, University of Liverpool, Liverpool, UK; 2 University of Central Lancashire, Preston, UK; 3 Centre for Local Economic Strategies, Manchester, UK

**Keywords:** EMPLOYMENT, DISABLED PERSONS, ETHNIC GROUPS, Health inequalities

## Abstract

**Background:**

People-centred initiatives to create and retain local wealth, such as Community Wealth Building (CWB), have potential to stimulate regional economic regeneration that addresses economic inequalities by increasing the economic inclusion of more disadvantaged groups. Preston, a relatively deprived city in England, has implemented a CWB programme that has been associated with improvements in local wages and well-being. We estimated the effect of Preston’s CWB programme on employment and examined differential effects by disability status and other equality dimensions.

**Methods:**

We conducted a difference-in-differences analysis combined with entropy balancing to estimate the effect of the introduction of the CWB programme in Preston on local employment rates, using individual-level data from the Annual Population Survey collected between 2011 and 2019. We performed subgroup analysis to investigate whether the effect on employment was modified by disability, ethnic group, sex or education level.

**Results:**

We analysed survey responses from 95 476 individuals. The introduction of the CWB programme was associated with an increase in the employment rate of 4% (95% CI 2.4% to 5.7%) among people living in Preston, compared with what would have been expected in the absence of the programme. The effect on employment was greater among people with disabilities, minority ethnic groups, men and people with lower levels of education.

**Conclusions:**

Our findings indicate that CWB can have a positive impact on employment over a relatively short period of time, which disproportionately benefits people with disabilities and other disadvantaged groups. This evidence can be used to inform the development, implementation and evaluation of CWB strategies in other places. Preston’s CWB programme may represent a strategy to achieve more equitable economic growth and reduce health inequalities.

**WHAT IS ALREADY KNOWN ON THIS TOPIC**

- Our previous ecological analysis found that the introduction of Preston’s Community Wealth Building (CWB) programme was associated with improvements in local wages and well-being that were greater than expected compared with other similar places. The analysis used publicly available Annual Population Survey data at local authority level, and the results indicated the introduction of Preston’s CWB programme was associated with a 4.7% increase in the employment rate in Preston, which did not reach statistical significance. We were unable to investigate differences in effect between different groups within Preston.

**WHAT THIS STUDY ADDS**

- We conducted a more robust analysis to examine the effect of Preston’s CWB programme on employment using individual-level data from the Annual Population Survey. This enabled us to control for individual-level confounding, attain greater precision of the effect estimates due to the large number of individual survey responses included in the analysis, and analyse differential employment effects across equality groups, including by disability. Using difference-in-differences methods combined with entropy balancing, we found that following the introduction of the programme, employment rates increased by 4% (95% CI 2.4% to 5.7%) among people living in Preston compared with what would have been expected in the absence of the programme. We found that the employment gains associated with the programme were greatest among more disadvantaged groups, that is, people with disabilities, minority ethnic groups, and people with lower levels of education.

**HOW THIS STUDY MIGHT AFFECT RESEARCH, PRACTICE OR POLICY**

- Our research provides valuable evidence on alternative place-based approaches to economic development, centred around economic democracy and social value. Our findings indicate that the CWB programme in Preston has differential impacts across multiple equality dimensions, reducing the disability employment gap, which has implications for policies that aim to promote inclusive economic change that supports health and well-being.

## Introduction

 The UK is one of the most regionally unequal economies in the industrialised world.^1^ Despite efforts by successive UK governments to tackle the issue, substantial disparities in productivity, wealth, resources and living standards persist between UK regions.^2 3^ Poorer health, high levels of disability and lower employment of disabled people in more disadvantaged regions are a major contribution to these economic inequalities.^4^ Furthermore, evidence suggests these inequalities have widened over recent decades,^3^ which underscores the need for innovative approaches to stimulate economic development in disadvantaged places.

Novel, people-centred initiatives to create and retain local wealth, such as Community Wealth Building (CWB), have garnered much interest because theoretically they have considerable potential to stimulate regional economic regeneration to address economic differences between and within places.^5 6^ Preston, a city in the North West of England, has led the way in developing a CWB programme which aims to create a resilient and inclusive economy for the benefit of the local area. This multicomponent programme initially leveraged the influenceable spend of anchor institutions within Preston to support the development of local enterprises, invest local wealth into the local economy, improve recruitment and employment conditions, and maximise socially productive use of land and property. Our previous ecological analysis found that the introduction of Preston’s CWB programme was associated with improvements in local wages, well-being and employment that were greater than expected compared with other similar places.^7^

Higher levels of disability and lower levels of educational attainment contribute to the economic differences between more disadvantaged places such as Preston and the rest of the UK, in addition to lower employment rates among people with disabilities and less education. The disability employment gap has remained persistently large, despite ongoing government strategies to reduce it.^8^ Previous strategies have tended to focus on improving the employability of disabled people, and less attention has been paid to the benefits of supply-side approaches that increase available jobs in places with high levels of disability.^9 10^ It is important, therefore, to understand whether CWB initiatives reduce inequalities within places by disproportionately benefiting more disadvantaged groups by, for example, having a greater impact on the employment of people with disabilities and people with lower educational attainment.

Previous analysis of the employment effects of the Preston CWB initiative used publicly available survey data aggregated at local authority level, which presented estimation complications since there was only one local authority intervention unit of interest (Preston). In our previous study, it was not possible to investigate the differential effect of CWB by equality groups, to understand whether the programme improved economic inclusion within Preston. For this current study, we accessed secure versions of the survey data to examine the effect of Preston’s CWB programme on employment using individual-level data, enabling increased robustness to ecological bias, greater precision and evaluation of differential effects across population subgroups.

This study had two aims. The first was to estimate the effect of the introduction of Preston’s CWB programme on employment rates, while controlling for individual-level covariates including age, sex and education level. The second was to investigate whether CWB reduced inequalities in employment and increased economic inclusion within Preston by investigating effects for subgroups defined by disability, ethnicity, sex and education level.

## Methods

### Setting and intervention

The city of Preston has a population of around 140 000 and is within the 20% most deprived local authorities in England based on the Indices of Multiple Deprivation.^11^ In Preston, 28% of the working age population have a disability, compared with 25% in England as a whole, and 53% of these people are employed, compared with 57% nationally.^12^ Work on CWB in Preston started in 2012, with Preston City Council becoming the first local authority in the north of England to become accredited as a Living Wage Employer by the Living Wage Foundation, which sets a minimum wage standard that accredited employers agree to pay.^13^ Through a series of activities with officials and procurement leads, including running workshops to identify the behaviours and patterns which influenced procurement, several anchor institutions within Preston agreed to use their influenceable spend for CWB. This led to a shift in their spending towards local and socially responsible suppliers.^14^ For this study, we take 2015 as the start date of the intervention, as this is the point from which there had been some change in procurement that could realistically have economic effects based on previous analysis of procurement spend retained within Preston.^14^ Work started in 2017 to support the development of local worker-owned businesses with the establishment of Preston Cooperative Development Network^15^ and the funding of local worker-owned businesses. To date, the development of new cooperatives has been modest but includes the establishment of the cooperatively organised Preston Cooperative Education Centre. The development of cooperatives as an objective of CWB aligns with an aspiration to expand employment and grow decent work.^16^

### Data sources and measures

We analysed data from the Annual Population Survey (APS),^17^ which is conducted by the Office for National Statistics (ONS) and aims to provide estimates between censuses of social and labour market variables at a local area level.^18^ The APS uses data combined from two waves of the Labour Force Survey and data collected on a local sample boost to increase the sample size. The APS has the largest coverage of any household survey in the UK and is an ONS recommended source for employment-related statistics. We applied to the UK Data Service for permission to access Secure versions of APS datasets.

All individuals included in our analysis were aged between 16 and 64 years to represent those most likely to be economically active. Our outcome variable was employment, defined as survey respondents who reported being an employee or self-employed, reflecting those in paid work. This is the outcome that was most plausibly affected by the main component of CWB implemented at this time, that is, increased procurement from businesses within Preston and the surrounding areas. Subgroups of the population were defined by ethnic group, sex, highest educational qualification-level attained and disability. Disability was defined as those reporting health problems or conditions/illnesses lasting or expected to last 12 months or more, that affect the kind or amount of paid work they might do. We dichotomised the ethnicity and education-level subgroups because of the relatively small number of survey respondents in Preston (see online supplemental appendix 1 for further details of measures).

### Design and analysis

We used difference-in-differences methods combined with entropy balancing to estimate the effect of the introduction of the CWB programme in Preston on local employment rates. This was calculated as the difference between the change in employment rates among survey respondents residing in Preston (intervention group) and the change in employment rates among survey respondents residing in comparison areas across England (comparison group), before and after the introduction of the intervention. We used the same comparison areas as our previous analysis, defined as all lower tier local authorities in the North or Midlands, with a population between 90 000 and 250 000, that are within the 25% most deprived local authorities in England, and are not already developing CWB programmes.^7^ This gives 16 local authorities that were used to construct the comparison group (online supplemental appendix 2).

Difference-in-differences methods control for measured and unmeasured time-invariant differences between the intervention and comparison groups, as well as time-varying factors that affect the outcome in the same way between the groups.^19^ The main assumption of difference-in-differences analysis is the parallel trends assumption. If the trend in the outcome in the intervention and comparison groups would have been parallel in the absence of the intervention, then the difference between the change in the outcomes between the two groups provides an unbiased estimate of the intervention’s effect.^20^ This assumption becomes more plausible if the intervention and comparison groups are similar to each other in terms of trends in the outcome in the preintervention period.

We, therefore, used entropy balancing to reweight the intervention and comparison groups to achieve perfect balance on specified moments of a set of covariates.^21 22^ We balanced on preintervention outcome trends^23^ and individual-level covariates, age, sex and education level. Due to the cross-sectional nature of the surveys, it was not possible to calculate outcome trends per individual, and therefore, we used linear regression to estimate preintervention trends of annual averages of employment rates per local authority. We also balanced on average local authority outcome values in 2015. The R WeightIt package^24^ was used to estimate weights for the comparison group using entropy balancing. These weights were included in a linear regression model of the employment outcome with an intervention group by pre/postperiod interaction term to estimate the effect of the introduction of the intervention, and SEs clustered by local authority.

We performed subgroup analysis to investigate whether this association was modified by respondents’ disability status, ethnic group, sex or education level. We reweighted the intervention and comparison groups for each subgroup to achieve balance across the covariates (as above), except we did not balance on education level or sex when examining these subgroups, respectively. We investigated the parallel trends assumption using graphical methods and regression models to compare trends in outcomes between the intervention and comparison groups in the preintervention period. Analyses were conducted using R (V.4.3.2) within the UK Data Service SecureLab.

## Results

**Figure 1:**
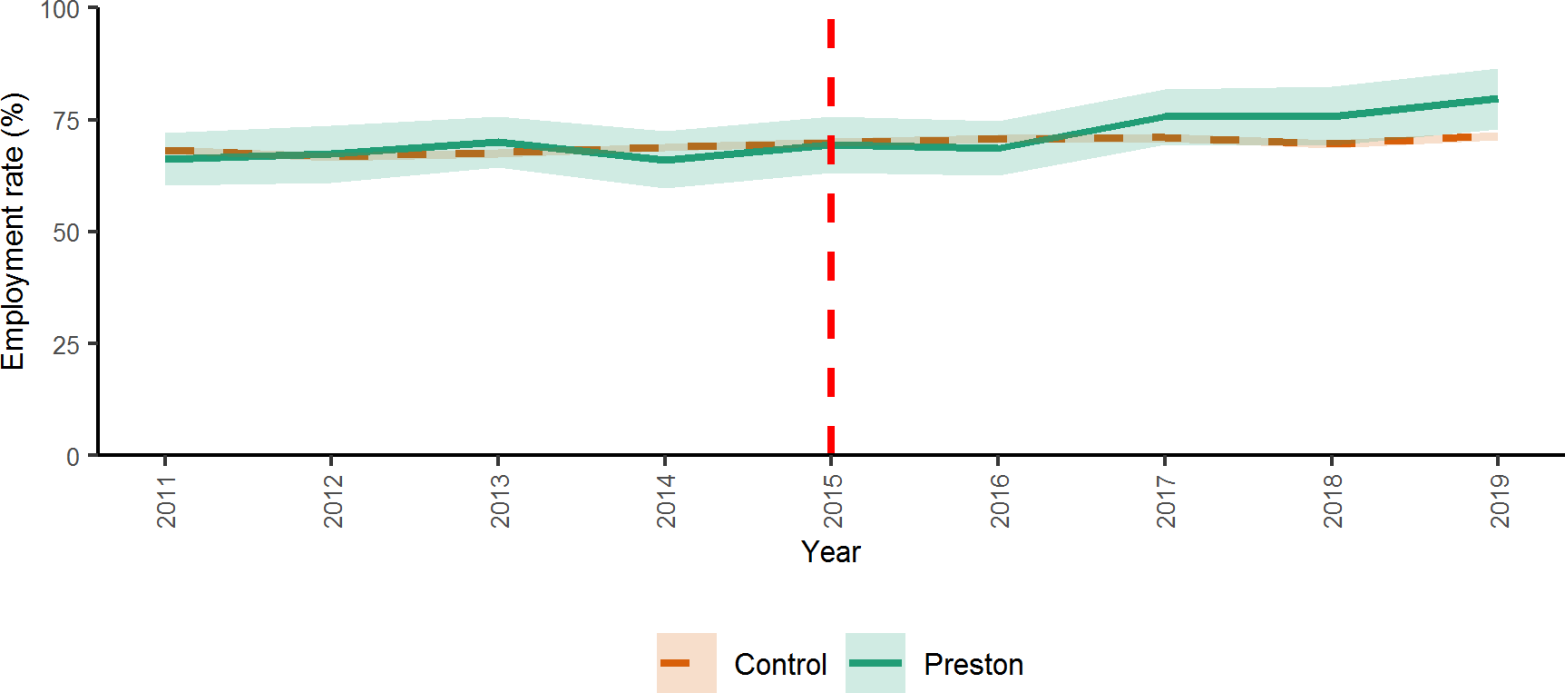
Trends from 2011 to 2019 in employment rates in the intervention and comparison groups, before and after the introduction of the intervention. Shaded areas around the lines represent the 95% CIs.

In total, 95 476 individual survey responses were available to analyse. Figure 1 indicates how employment rates changed in Preston after 2015 compared with what would have been expected given trends in employment rates in other similar areas. Trends in employment rates were similar before 2015 between the intervention and comparison groups. Following the introduction of the CWB programme, employment rates among the intervention group increased to a greater extent compared with the comparison group.

The results from the difference-in-differences analysis indicate there was a statistically significant increase in employment rates in the intervention group relative to the comparison group after 2015. The analysis shows that the introduction of the intervention was associated with an increase in the employment rate of 4% (95% CI 2.4% to 5.7%) among people living in Preston, compared with what would have been expected in the absence of the intervention (see online supplemental appendix 3 for full model results).

In the stratified analysis, we see a greater increase in the employment rate associated with the introduction of the intervention for those with a disability that affects the amount or kind of paid work they might do, compared with those without any long-term health problems or disabilities (table 1). When the analysis was stratified by ethnic groups, there was a greater increase in the employment rate associated with the introduction of the intervention for black and minority ethnic groups compared with white ethnic groups. Additionally, there was a greater increase in the employment rate associated with the introduction of the intervention for people with no or secondary educational qualifications, compared with people with a degree or tertiary educational qualification. Finally, there was a greater increase in the employment rate associated with the introduction of the intervention for men compared with women. Full model results are provided in online supplemental appendix 3.

**Table 1:** Estimates of the effect of the intervention on the employment rate outcome from the difference-in-differences regression models stratified by ethnic group, education level, sex and disability status

Subgroup	Absolute effect on employment rate of 16–64 years (%)	95% CI (lower, upper)	P value
Ethnic group (black and minority ethnic)	6	(0.5 to 11.5)	0.036
Ethnic group (white)	3.6	(1.5 to 5.7)	0.002
Education level (none or secondary)	5	(3.1 to 6.9)	<0.001
Education level (degree or tertiary)	0.1	(−0.8 to 1)	0.797
Women	1.7	(0.1 to 3.3)	0.034
Men	5.8	(4.6 to 7)	<0.001
Those with a disability affecting amount of paid work possible	16.4	(13.5 to 19.4)	<0.001
Those with a disability affecting kind of paid work possible	22.1	(15.1 to 29.2)	<0.001
Those with no long-term health problems or disabilities	2.9	(1.6 to 4.2)	<0.001

See online supplemental appendix 3 for full model results.

## Discussion

This study evaluated the impact of Preston’s CWB programme on employment and explored differential effects by equality dimensions (disability, ethnicity, sex and education level), using natural experimental methods. We found that following the introduction of the programme, employment rates increased among people living in Preston compared with what would have been expected in the absence of the programme. The effect on employment was greater among people with disabilities, minority ethnic groups, men and people with lower levels of education.

A central component of CWB is harnessing the spending power of anchor institutions to support the development of local supply chains.^25^ In 2013, Preston City Council and the Centre for Local Economic Strategies (CLES) conducted analysis showing that only 5% (£38 million) of anchor institution spend was with organisations based in Preston.^14^ Following the introduction of CWB, the spend analysis was repeated, revealing that spend of anchor institutions retained within Preston had increased to £112 million in 2017.^14^ It is highly plausible that redirecting wealth back into the local economy and increasing the revenue of local firms would stimulate local business innovation and capacity, and have a positive impact on local employment.^26^ Additionally, our results were similar in magnitude to a previous evaluation of CWB in Preston using aggregate local authority-level data. Our previous analysis found that the introduction of Preston’s CWB programme was associated with a 4.7 percentage point increase in the employment rate in Preston compared with a synthetic counterfactual, which did not reach statistical significance.^7^

This study had a number of strengths. Compared with our previous analysis of local authority-level data, using individual-level survey data allowed us to conduct an analysis that was more robust since we were able to control for individual level confounding, and our effect estimates were likely more precise due to the large number of individual survey responses included in our analysis (N=95 476). We balanced the intervention and comparison groups on potential confounding variables and trends in employment prior to the intervention, and this was confirmed by the parallel nature of the trends in the employment rate before the intervention. The difference-in-differences analysis would have also effectively controlled for all time-invariant differences between the intervention and comparison groups, as well as time-varying factors that affected employment rates in the same way between the groups.

It is, however, difficult to rule out the possibility that different trends in unobserved confounding factors between the intervention and comparison groups could have influenced the results. For example, there may have been concurrent economic changes in Preston that contributed to increases in employment, such as the University of Central Lancashire (UCLAN) investment of £200 million for its Preston campus in 2015. However, as UCLAN is one of the anchor institutions involved in the CWB programme, its adoption of a social value framework for this investment may have enhanced its beneficial impact on the economy. We also appreciate the heterogeneity within the ethnicity and education level subgroups, and while it would have been preferable to analyse non-white ethnic groups separately, this was not possible due to the small number of respondents within these categories. Our subgroup analysis has limitations in terms of statistical power and does not allow for direct comparison on effect sizes between groups, as we estimated separate models for each subgroup rather than using three-way interactions. This, however, enabled reweighting the intervention and comparison groups for each subgroup to achieve balance across the covariates within subgroups, achieving conditional preintervention parallel trends within subgroups. This mitigated bias that could result from an interaction analysis where there were violations of parallel trends within subgroups.

### Implications for policy

Many places across the UK are developing and implementing CWB strategies. For example, the Scottish government has developed a CWB strategy^27^ as a key part of their National Strategy for Economic Transformation.^28^ The government of Wales has developed a procurement framework focusing on the retention and use of local wealth,^29^ and CLES is partnering with the Welsh Government to support anchor institutions in selected Public Services Board areas to explore CWB approaches, with a focus on progressive procurement. CLES is also working with more than 15 local authorities across England and Scotland to support local CWB strategies. Furthermore, the National Health Service (NHS) has recognised that, as a major anchor institution, it has a role in CWB and has established the Health Anchors Learning Network.^30^

Our research provides evidence of the impact of Preston’s CWB programme which can be used to inform the development, implementation and evaluation of CWB strategies in other places. Our findings indicate that CWB can have a positive impact on employment over a relatively short period of time, and that it is possible to monitor economic progress on the scale of a small city using robust controlled methods. When available, results from similar evaluations by other places implementing CWB strategies will be crucial to understanding the full potential of CWB as a policy tool.

We found that employment gains associated with the introduction of Preston’s CWB programme were greatest among more disadvantaged groups, that is, people with disabilities, minority ethnic groups and people with lower levels of education. While further research is needed to unpick the explanations for these findings, it is possible that the core principles of Preston’s CWB programme linked to creating a more inclusive and fairer economy have increased focus among anchor institutions on social value procurement and recruitment that has benefited more disadvantaged groups. The jobs created by these policies may be more accessible for more disadvantaged groups, particularly if they are introduced in places with high numbers of people with disabilities and lower levels of education. While research shows that historically the loss of jobs from disadvantaged places in the UK due to deindustrialisation has contributed to high levels of people out of work due to disability,^31^ there has been limited research into whether interventions promoting jobs growth in these communities disproportionately benefit people with disabilities. Our study suggests that CWB may have positive benefits in reducing the disability employment gap. In addition to adversely affecting the lives of people with disabilities, low employment among disabled people is a major cost to public finances. Welfare spending on disability is currently £40 billion^32^ and has increased markedly in recent years. Approaches such as CWB, which increase employment of disabled people, could therefore also offer significant cost savings to the UK treasury.

Economic improvements that disproportionately benefit more disadvantaged groups are also more likely to lead to population health benefits and reduced health inequalities.^33 34^ Indeed, our previous ecological analysis found that reductions in depression prevalence associated with the CWB programme appeared to be greater in the most deprived areas of Preston. Together, these findings suggest Preston’s CWB programme presents a practical approach to achieve more equitable economic growth and reduced health inequalities, which may be of interest to national and local governments aiming to deliver more inclusive economic development that promotes well-being.

## Supplementary material

10.1136/jech-2024-223499 online supplemental file 1

## Data Availability

Data may be obtained from a third party and are not publicly available.
