# Supplementary material for: Understanding the differential effects on employment of a community wealth building programme in England: a difference-in-differences study
Source: J Epidemiol Community Health. 2025 May 16;79(9):e223499. doi: 10.1136/jech-2024-223499 (PMC12418530; doi:10.1136/jech-2024-223499)
Supplement: online supplemental file 1 [file jech-79-9-s001.pdf]

Supplementary file

Table of contents

Appendix 1.	Measures derived from the Annual Population Survey	**2**
Appendix 2.	Comparison areas	**3**
Appendix 3.	Full model results	**4**

Appendix 1. Measures derived from the Annual Population Survey (APS)

Age	Respondents aged 16 to 64 years were included in this analysis
Sex	Question – What is your current sex?<br>1) Male, or<br>2) Female
Disability	Question – Do you have any physical or mental health conditions or illnesses lasting or expecting to last 12 months or more? Yes, or No<br><br>Question – Does this health problem affect the kind of paid work that you might do? Yes, or No<br><br>Question – Does this health problem affect the amount of paid work that you might do? Yes, or No
Ethnic group	Question – What is your ethnic group?<br>Categorised into two groups for this analysis due to small sample sizes in Preston:<br>1) White, or<br>2) Mixed/Multiple ethnic groups, or Asian/Asian British, or Black/African/Caribbean/Black British, or Chinese, or Arab, or Other ethnic group
Education level	Derived variable – Highest qualification<br>Categorised into two groups for this analysis due to small sample sizes in Preston:<br>1) Degree or equivalent, or Higher education<br>2) GCE, A-level or equivalent, or GCSE grades A*-C or equivalent, or Other qualifications, or No qualification
Employment	Derived variable – Basic economic activity<br>We defined employment as being an employee or self-employed. Those in government employment and training programmes, unpaid family workers, the unemployed as defined by the ILO and inactive, were not classed as being in employment for this study.

Appendix 2. Comparison areas - lower tier local authorities in the North or Midlands, with a population between 90,000 to 250,000, that are within the 25% most deprived local authorities in England, and are not already developing CWB programmes

Local Authority
Middlesbrough
Redcar and Cleveland
Stockton-on-Tees
Halton
Blackburn with Darwen
Telford and Wrekin
Chesterfield
Pendle
East Lindsey
Lincoln
Ashfield
Mansfield
Knowsley
St. Helens
Barnsley
Calderdale

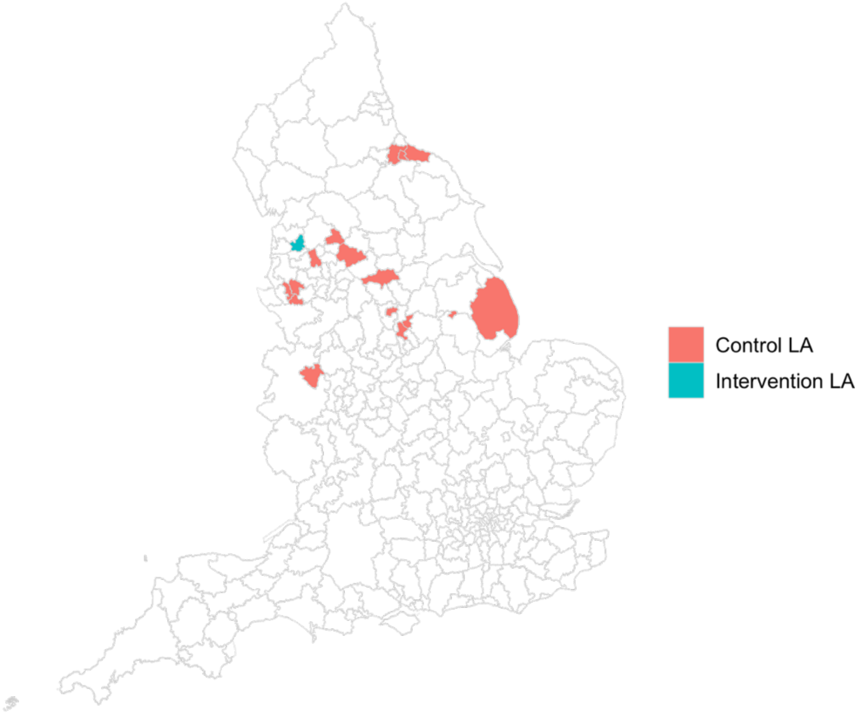

The city of Preston is located within the North West region of England, has a population of approximately 140,000 and is within the 20% most deprived local authorities in England based on the Indices of Multiple Deprivation. The comparison local authority areas were approximately matched to Preston based on geographic location (North or Midlands), population (between 90,000 to 250,000), and level of deprivation (within the 25% most deprived local authorities in England). It was also important that our control areas were not already developing CWB programmes.

Appendix 3. Full model results

Estimates from the difference-in-differences regression model indicating the change in employment in the intervention group before (2011-2015) and after (2016-2019) the start of the CWB programme, compared to the change in employment between the same time periods in the comparison group.

	Effect on Employment rate
(Intercept)	68.252
	[66.587, 69.916]
	p value = <0.001
Period	2.446
	[0.770, 4.122]
	p value = 0.007
Intervention	-0.439
	[-2.103, 1.226]
	p value = 0.584
Period:Intervention	4.043
	[2.367, 5.718]
	p value = <0.001
Num.Obs.	95476
R2	0.001
R2 Adj.	0.001
AIC	1004730.5
BIC	1004768.4
RMSE	46.65

Estimates of the effect of the intervention on the employment rate outcome from the difference-in-differences regression models stratified by ethnic groups

	Subgroup (Ethnic group = BAME)	Subgroup (Ethnic group = White)
(Intercept)	56.122	70.347
	[49.208, 63.036]	[68.307, 72.387]
	p value = <0.001	p value = <0.001
Period	1.964	2.901
	[-3.538, 7.466]	[0.824, 4.979]
	p value = 0.460	p value = 0.009
Intervention	0.774	0.207
	[-6.140, 7.689]	[-1.833, 2.246]
	p value = 0.815	p value = 0.833
Period:Intervention	5.954	3.621
	[0.452, 11.456]	[1.544, 5.698]
	p value = 0.036	p value = 0.002
Num.Obs.	7416	85221
R2	0.001	0.001
R2 Adj.	0.000	0.001
AIC	78991.6	895196.4
BIC	79019.3	895233.8
RMSE	49.71	46.21

Estimates of the effect of the intervention on the employment rate outcome from the difference-in-differences regression models stratified by disability status

	Subgroup (Health problem affects amount of paid work: Yes)	Subgroup (Health problem affects kind of paid work: Yes)	Subgroup (No health problems)
(Intercept)	30.881	36.813	73.937
	[27.232, 34.531]	[33.891, 39.735]	[71.087, 76.788]
	p value = <0.001	p value = <0.001	p value = <0.001
Period	3.778	-0.355	2.874
	[0.793, 6.763]	[-7.422, 6.713]	[1.582, 4.165]
	p value = 0.016	p value = 0.917	p value = <0.001
Intervention	-8.005	-10.069	1.187
	[-11.655, -4.356]	[-12.991, -7.146]	[-1.664, 4.037]
	p value = <0.001	p value = <0.001	p value = 0.390
Period:Intervention	16.436	22.140	2.894
	[13.451, 19.421]	[15.072, 29.208]	[1.603, 4.185]
	p value = <0.001	p value = <0.001	p value = <0.001
Num.Obs.	16161	18005	61161
R2	0.002	0.001	0.001
R2 Adj.	0.002	0.001	0.001
AIC	169294.9	189863.9	632252.5
BIC	169325.6	189895.1	632288.6
RMSE	45.54	47.15	42.51

Estimates of the effect of the intervention on the employment rate outcome from the difference-in-differences regression models stratified by education level

	Subgroup (Education level: Higher education)	Subgroup (Education level: A-level or lower)
(Intercept)	80.982	61.475
	[78.675, 83.289]	[59.459, 63.491]
	p value = <0.001	p value = <0.001
Period	0.288	2.439
	[-0.609, 1.185]	[0.529, 4.349]
	p value = 0.506	p value = 0.016
Intervention	2.619	-0.156
	[0.312, 4.926]	[-2.172, 1.860]
	p value = 0.029	p value = 0.871
Period:Intervention	0.111	4.979
	[-0.786, 1.007]	[3.068, 6.889]
	p value = 0.797	p value = <0.001
Num.Obs.	25514	67657
R2	0.000	0.001
R2 Adj.	0.000	0.001
AIC	257629.2	717185.1
BIC	257661.8	717221.6
RMSE	37.70	48.48

Estimates of the effect of the intervention on the employment rate outcome from the difference-in-differences regression models stratified by sex

	Subgroup (Sex: Female)	Subgroup (Sex: Male)
(Intercept)	63.870	71.994
	[61.967, 65.772]	[69.937, 74.052]
	p value = <0.001	p value = <0.001
Period	2.861	2.766
	[1.267, 4.455]	[1.565, 3.967]
	p value = 0.002	p value = <0.001
Intervention	-0.425	0.218
	[-2.328, 1.477]	[-1.839, 2.275]
	p value = 0.642	p value = 0.825
Period:Intervention	1.738	5.758
	[0.144, 3.333]	[4.557, 6.959]
	p value = 0.034	p value = <0.001
Num.Obs.	50368	45108
R2	0.001	0.001
R2 Adj.	0.001	0.001
AIC	532882.8	470796.9
BIC	532918.1	470831.7
RMSE	47.98	44.68
